# An ontogenetic approach to gynecologic malignancies

**DOI:** 10.1007/s13244-016-0480-y

**Published:** 2016-04-15

**Authors:** Inês A. Santiago, António P. Gomes, Richard J. Heald

**Affiliations:** Radiology Department, Champalimaud Foundation, Av. Brasília, 1400-038 Lisbon, Portugal; B Surgery Department, Hospital Fernando Fonseca, E.P.E., IC19, 2720-276 Amadora, Portugal; Colorectal Surgery Department, Champalimaud Foundation, Av. Brasília, 1400-038 Lisbon, Portugal

**Keywords:** Ontogenetic anatomy, Ontogenetic surgery, Gynaecologic cancer, Total mesometrial excision, MR imaging

## Abstract

**Abstract:**

Ontogenetic anatomy is the mapping of body compartments established during early embryologic development, particularly well demarcated in the adult pelvis. Traditional cancer surgery is based on wide tumour excision with a safe margin, whereas the ontogenetic theory of local tumour spread claims that local dissemination is facilitated in the ontogenetic compartment of origin, but suppressed at its borders in the early stages of cancer development. Optimal local control of cancer is achieved by whole compartment resection with intact margins following ontogenetic “planes”. The principles embodied in this hypothesis are most convincingly supported by the results of the implementation of total mesorectal excision in rectal cancer, and more recently, by innovative surgical approaches to gynaecologic malignancies. The high resolution contrast of MR, accurately delineating pelvic fascial compartments, makes it the best imaging modality for gynaecologic cancer surgery planning following these principles, but requires interpretation of imaging anatomy from a different perspective.

***Teaching Points*:**

• *Ontogenetic anatomy refers to mapping of embryologically determined body compartments*.

• *Ontogenetic theory claims tumour growth is not isometrical, but rather compartment permissive*.

• *Ontogenetic principles are highly supported by the outcome results of total mesorectal excision*.

• *Innovative gynaecologic cancer surgery approaches based on ontogenetic theory show promising results*.

## Introduction

Ontogenetic anatomy is based on the establishment of compartments during early embryologic development by lack of mixing of proliferating groups of cells—anlagen or primordia—at demarcated boundaries, which can be identified at certain sites in the adult body, for example the pelvis [1].

Traditional concepts of local tumour spread based on isotropic tumour extension led to metrically defined margins of resection for standard surgical procedures [1].

Ontogenetic compartment theory states local tumour spread is facilitated in the permissive ontogenetic compartment, but suppressed at its borders; therefore, requiring its complete resection with intact margins, irrespective of margin width, for local disease control [1]. Its validity was established for rectal cancer long before its description, with the implementation of total mesorectal excision which improved local control far more than had been anticipated [2, 3]. It is supported by pattern analysis of cancers of the lower genital tract, as well as total mesometrial resection in cervix cancer and ontogenetic surgery for vaginal and vulvar malignancies [4–13].

MR imaging has already been established as the method of choice for local staging in rectal and gynaecologic cancer due to its high contrast resolution. It allows not only tumour localization and delineation with great precision, but also compartment delimitation through the identification of pelvic fascia and ligaments that constitute most of the ontogenetic compartment boundaries of the adult pelvis. Its relevance with respect to patient management in gynaecologic cancer surgery is, therefore, expected to increase.

In the first part of the present paper, authors describe the principles of ontogenetic anatomy, relevant early embryology, the principles of ontogenetic theory of local tumour spread and current evidence supporting it. The second part of the paper corresponds to the reinterpretation of gynaecologic anatomy based on MR imaging, as relevant for oncologic surgery planning and in accordance with the theory principles, with some added example cases.

## Ontogenetic compartmentalization

### Early embryology

**Fig. 1:**
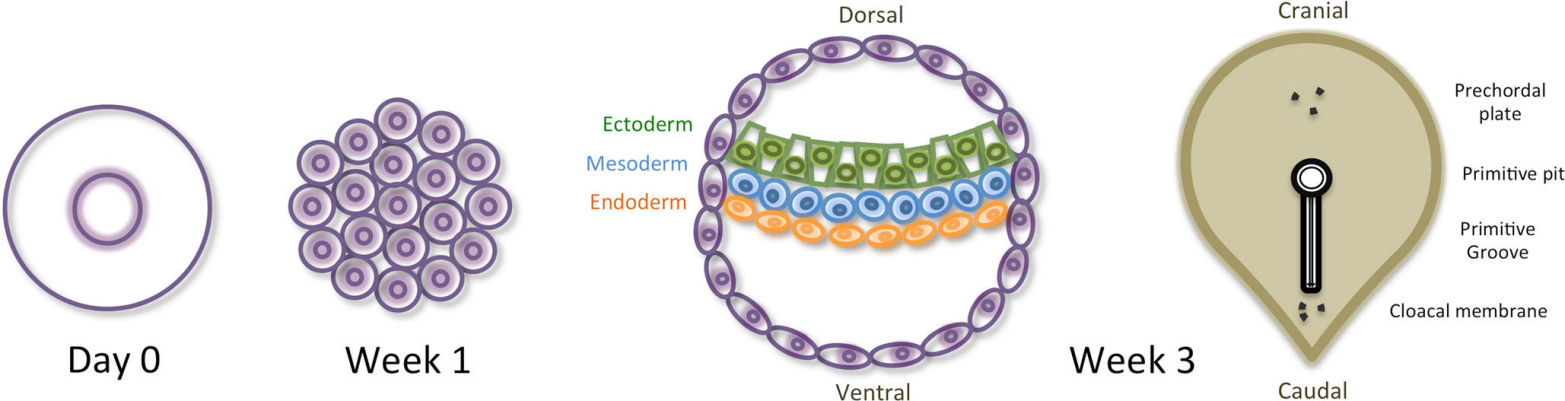
Early embryology. **Day 0**: The zygote results from the fusion of the spermatozoon and the oocyte II; **Week 1:** Cell division without volume expansion takes place during the 1st week forming a structure which resembles a mulberry—the morula. It will originate not only the embryo but also the membranes, placenta, and associated structures. Intense cell division and reorganization will then transform the morula into a single-layered hollow sphere—the blastula; **Week 3:** *Left*: Some blastomers move to a more interior location, forming a three-layered central disk during a process called gastrulation; *right:* craniocaudal polarity becomes apparent in the gastrula stage [14]

During the first week of embryologic development, the zygote evolves into a blackberry-like structure (morula). Cavitation and migration then accompany cell division, converting it into a cystic structure traversed by a tri-layered cell disk. During gastrulation, a clear anteroposterior and craniocaudal polarity of the embryo becomes apparent (Fig. 1) [14].

**Fig. 2:**
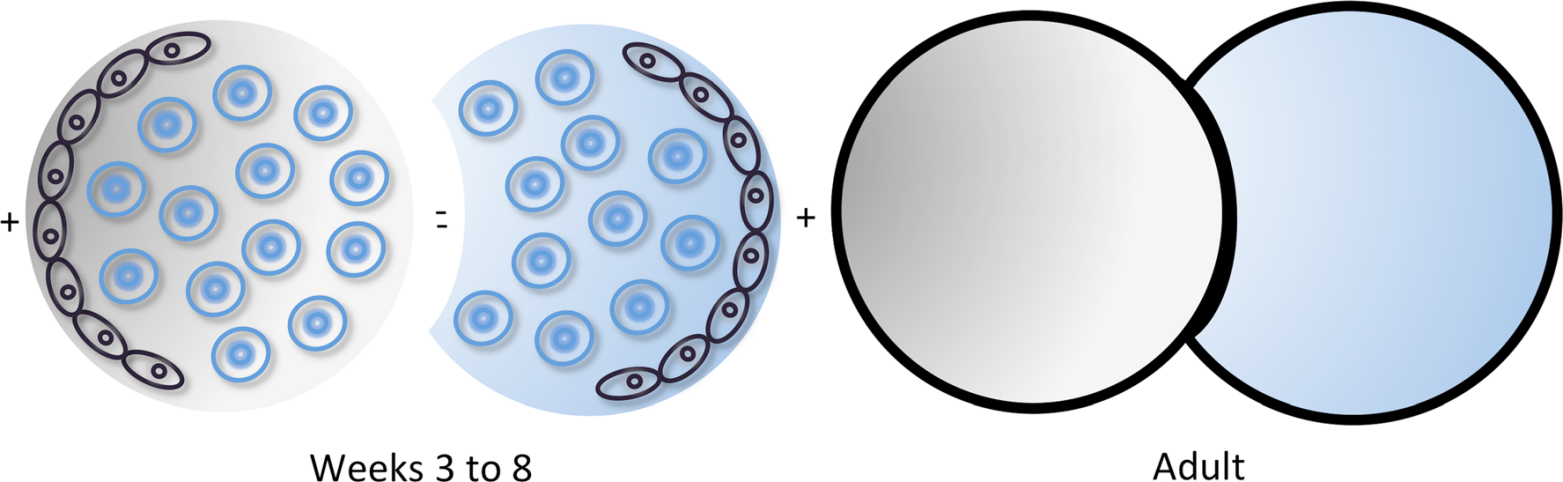
Organogenesis. **Weeks 3 to 8**: Primordia require the interaction between mesoderm-derived mesenchyme (*blue cells*) and ecto/meso/endoderm-derived epithelium (*black cells*); each primordium acts as a metabolic field driven by molecular signal gradients, constrained by neighbouring primordia; **Adult:** In the adult, primordia have become different ontogenetic compartments, adjacent to each other due to spacial body constraints [15]

Neurulation and organogenesis start taking place during weeks 3 to 8. Organogenesis requires the interaction between different tissues, usually mesoderm-derived mesenchyme and ecto/meso/endoderm-derived epithelium, forming anlagen or primordia. Primordia are the earliest tissue complexes with morphogenetic determination. They act as modified force fields driven by dynamic molecular signal gradients and subject to physical/spatial constraints (Fig. 2). Relative position of primordia with respect to the embryos' polarity determines their morphogenetic outcome [15].

### Principles of ontogenetic anatomy

Ontogenetic anatomy maps adult tissues with respect to their developmental origin, the anlagen or primordia, which function as independent modules of morphogenesis during embryonic and early fetal development. Lack of mixing of proliferating cells at the interface between different anlagen, established by molecular signal gradients, defines compartments (Fig. 2), subcompartments with different degrees of kinship being subsequently formed [7–12]. The margins of ontogenetic compartments may be more or less apparent in the adult. In the pelvis, they are quite easy to identify, namely using T2-weighted MR imaging, especially when fine slice small field specialized cuts are employed at optimal angles.

A classic functional anatomic unit such as an organ may derive from multiple anlagen and, therefore, be included in multiple ontogenetic compartments. Conversely, multiple functional anatomic units may originate in a single ontogenetic compartment [7–10, 12].

Ontogenetic compartments contain not only functional organs and structures, but also supporting tissue, often omitted in classic embryologic and anatomic descriptions. The borders between ontogenetic compartments may be delimitated by enveloping fascia, but may also be blurred due to epithelial coverage, focal fusion, or dense adherence [1, 8, 12].

### Ontogenetic compartment theory of local tumour spread

**Fig. 3:**
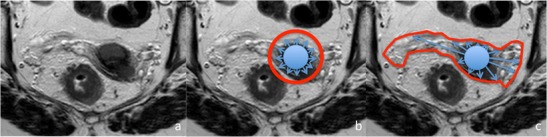
Axial high resolution T2-weighted image of the pelvis. **a** Fifty-six-year-old woman with synchronous malignancies of the rectum and cervix; **b** Centrifugal local tumour spread, irrespective of tissue boundaries, forms the basis of traditional cancer surgery, and dictates tumour excision with metrically defined margins; **c** According to the ontogenetic compartment theory of local tumour spread, tumour dissemination is not centrifugal, but rather confined to embryologically determined ontogenetic compartments until relatively late-stages. Resection along compartment borders is expected to lead to better disease control

Traditional principles of cancer surgery are based on centrifugal local tumour spread, irrespective of tissue boundaries (Fig. 3a and b). The margins required for an oncologic surgical specimen to be considered adequate sometimes lead to mutilating radical organ resection. Furthermore, despite adequate surgical technique with microscopic clear margins (R0), local recurrence rates in solid tumours still occur in 5 to 50 % of cases [16]. Adjuvant therapy has been recommended to a variety of them in order to reduce local recurrences, but leads to higher morbidity. Increasing the radial margin of resection has not always led to better disease control, particularly in highly prevalent malignancies such as breast and cervix [17–19].

The ontogenetic compartment theory of local tumour spread states that tumours initially spread within morphogenetically formed units, the compartment boundaries being tumour suppressive in the early stages of tumour growth. For transgression, phenotypic changes in tumour cells must occur, which then induce local permeation through inflammation or other phenomena. They are thought to occur relatively late in cancer progression and to favour compartments with a closer embryologic ancestry [1, 11]. As a result, in the early stages of the disease, resection along compartment borders leads to better disease control (Fig. 3c) [4].

### Current evidence

Since TME, which revolutionized rectal cancer management in 1982, embryology has been considered the key for defining the plane of dissection in oncologic surgery [20]. This was long before the principles of the ontogenetic theory of local tumour spread were first described by Hoeckel et al. in 2003. When compared to classic rectal cancer surgery, TME led to an increase in the 5-year survival rates from 30 % to 68 % and to a reduction in local recurrence rates from 30-50 % to 0-4 % [2, 3]. Also, incomplete mesorectal resection was associated with an increase in local and overall clinical recurrence compared to resection with an intact mesorectal fascia, mesorectal fat residues being the most plausible reservoir of viable residual tumour cells and still widely considered the single commonest cause of local recurrences [21–25].

Three trials based on the ontogenetic theory of local tumour spread using total mesometrial resection (TMMR) extended mesometrial resection (EMMR) and laterally extended endopelvic resection (LEER) for the treatment of cervix cancer, included a total of 367 patients stages I-B to II-B. These were associated with excellent outcomes with a locoregional tumour control rate at 5 years of 94 % [11]. Authors also proposed a new ontogenetic anatomy-based staging classification, found to be a better prognostic indicator of survival than classic pathological staging [11]. Ontogenetic anatomy-based surgical procedures were considered to have the potential to increase survival by 15 to 20 % compared to standard treatment in cervix cancer [8]. Pelvic topography of peak relapse probability after conventional radical hysterectomy for cervix cancer indicates that an incomplete resection of the corresponding ontogenetic compartment is the likely cause of recurrence [10, 11, 25].

A feasibility study for the application of robotic peritoneal mesometrial resection to intermediate/high risk endometrial cancer has also been published in 2013 concluding that is a feasible and safe technique [26].

In a series of 150 consecutive patients with distal vaginectomy as part of surgical treatment, 26 carcinomas of the lower genital track had infiltrated the distal vagina. Twenty-two out of those 26 tumours invaded the urethra/periurethral tissue (same ontogenetic compartment) while none out of the five carcinomas involving the dorsal wall invaded the mesorectum (different ontogenetic compartment) [12].

In a series of 54 consecutive patients with vulvar cancer, 46 tumours were locally confined to the ontogenetic compartment differentiated from the vulvar anlage. All eight tumours that transgressed into adjacent compartments exhibited signs of advanced malignant progression. Thirty-eight patients were treated based on the ontogenetic principles of cancer spread with compartment resection and anatomical reconstruction, and no local failures were found at a mean 19-month follow-up [13].

The principles of ontogenetic theory of local tumour spread also apply to extra-pelvic oncologic disease, namely to colon cancer, with the implementation of complete mesocolic excision (CME) [27–29]. CME has shown better outcomes relative to conventional surgery in stage I-III disease, with 4-year disease-free survival rising from 73.4 % to 85.8 % compared to conventional surgery and a global drop of 5-year local recurrence rates from 6.5 % to 3.6 % [28].

More recently, en bloc mesogastric excision was proved feasible for gastric cancer treatment, based on the same principles [30, 31]. Shinohara has applied the TME principle to upper GI surgery by identifying the “innermost dissectable layers”, which define the ontogenetic units developed from the upper gut [32].

The relevance of MR for compartment delineation and surgery planning is implicit in most of the publications concerning the ontogenetic anatomy-based approach to gynaecologic malignancies, cervix cancer in particular. However, image interpretation from an ontogenetic perspective requires a thorough rearrangement of relevant anatomic landmarks.

## Ontogenetic compartments in gynaecology

At 8 weeks of embryologic development, three morphologically distinct anlagen can be found in the female, which are involved in the formation of the lower genital tract (from cranial to caudal) [1].

### Paramesonephric-mesonephric-Mullerian tubercle complex

**Fig. 4:**
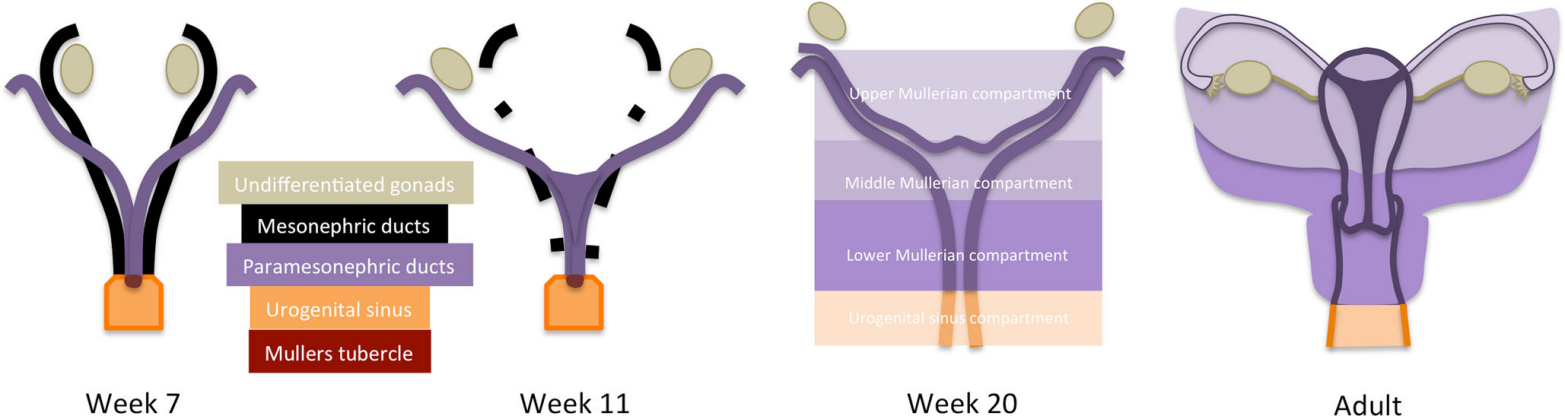
**Week 7:** Mullerian compartment structures originate from the Mullerian ducts, which arise as evaginations of the mesoderm lateral to the mesonephric ducts during the 7th week of gestation and gradually fuse medially; **Week 11:** Mesonephric ducts regress in the female; **Week 20 and adult:** The Mullerian compartment may be sub-divided in three sub-compartments. The ovaries, ovarian ligament, fimbriae, and distal vagina do not derive from the paramesonephric-mesonephric-Mullerian tubercle complex and are therefore not part of it. The lower Mullerian sub-compartment is sub-peritoneal [1, 8, 32]

**Fig. 5:**
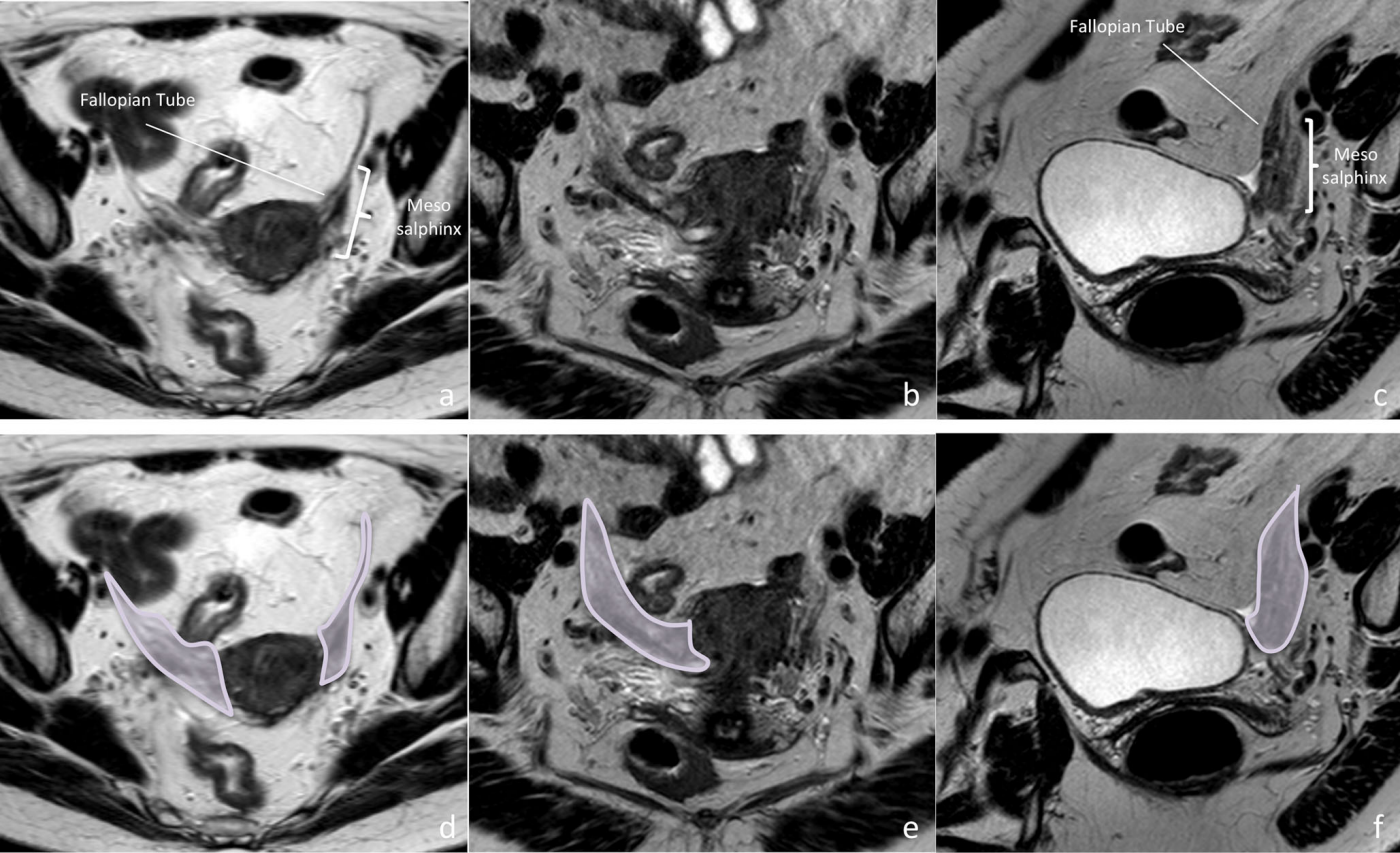
Structures contained in the upper Mullerian subcompartment and subcompartment boundaries. Upper Mullerian compartment contains tortuous tubular Fallopian tubes (above) enveloped by the mesosalpingia (upper portion of the broad ligaments). The latter contain vessels that extend between the uterine artery/vein medially and inferiorly and the ovarian artery/vein laterally and superiorly (at the suspensory ligament of the ovary). Compartment is limited inferiorly by the mesovary and proper ligament of the ovary. High resolution axial, coronal oblique, and sagittal oblique images (from left to right) depict subcompartment structures (**a**, **b,** and **c**) and boundaries (**d**, **e**, and **f**)

**Fig. 6:**
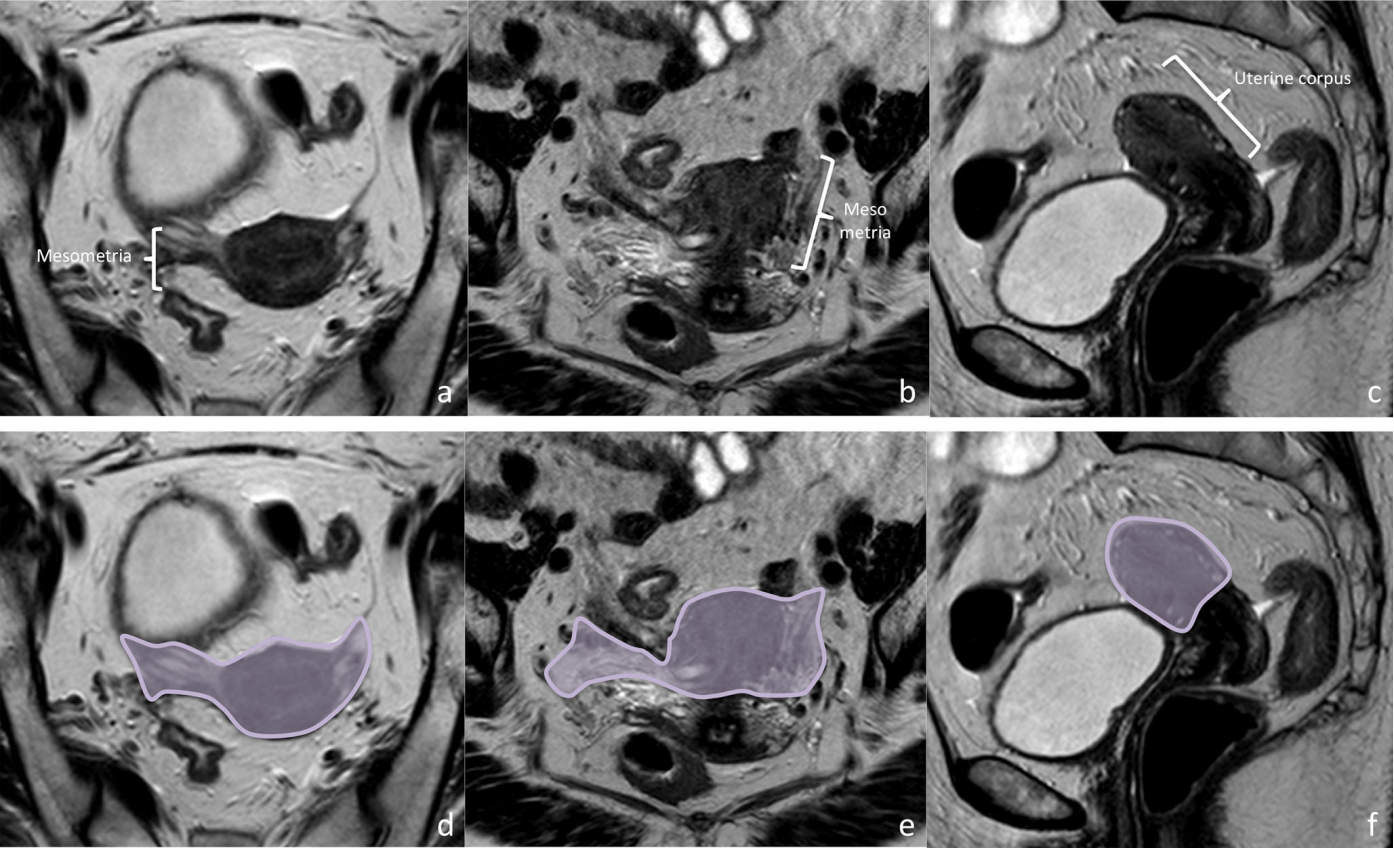
Structures contained in the middle Mullerian subcompartment and subcompartment boundaries. Middle Mullerian subcompartment contains the uterine corpus and the peritoneal mesometria (inferior portion of the broad ligaments). Uterine arteries and venous plexus course within the mesometria. High resolution axial, coronal oblique, and sagittal oblique images (from left to right) depict subcompartment structures (**a**, **b,** and **c**) and boundaries (**d**, **e**, and **f**)

**Fig. 7:**
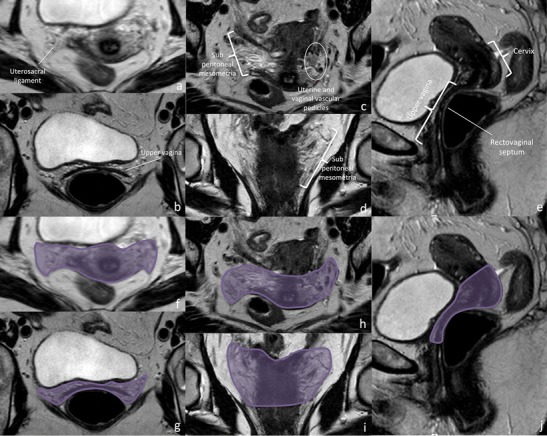
Structures contained in the lower Mullerian subcompartment and subcompartment boundaries. The lower Mullerian subcompartment contains the uterine cervix, the upper 2/3 to 3/4 of the vagina and the subperiotneal mesometria (which contains the uterine and vaginal vascular pedicles and autonomic nerve branches, the proximal part of the uterosacral ligaments, and the rectovaginal septum). Subperitoneum mesometria include the classically described parametria, which corresponds to the connective tissue that surrounds the supravaginal uterine cervix anteriorly and laterally). High resolution axial, coronal oblique and sagittal oblique images (from left to right) depict subcompartment structures (**a**, **b**, **c**, **d**, **e**) and boundaries (**f**, **g**, **h**, **i**, **j**)

**Fig. 8:**
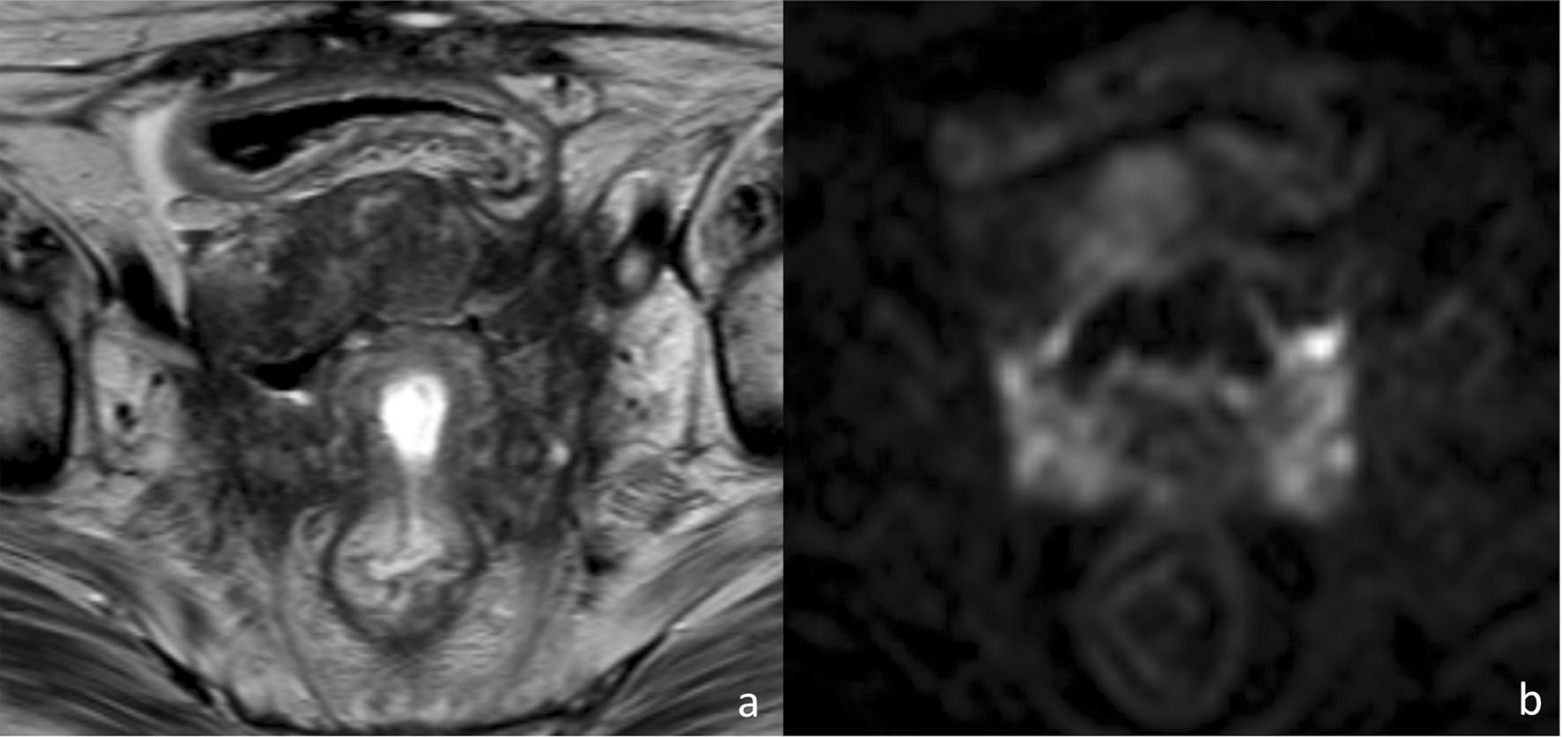
Fifty-two-year-old female patient with stage IVa cervical cancer after chemoradiation therapy, depicted in the axial plane, both on high-resolution T2-weighted **(a)** and diffusion-weighted **(b)** images. A clear preferential spread within the bow-tie-shaped Mullerian compartment, with extensive involvement of the mesometria, is seen

**Fig. 9:**
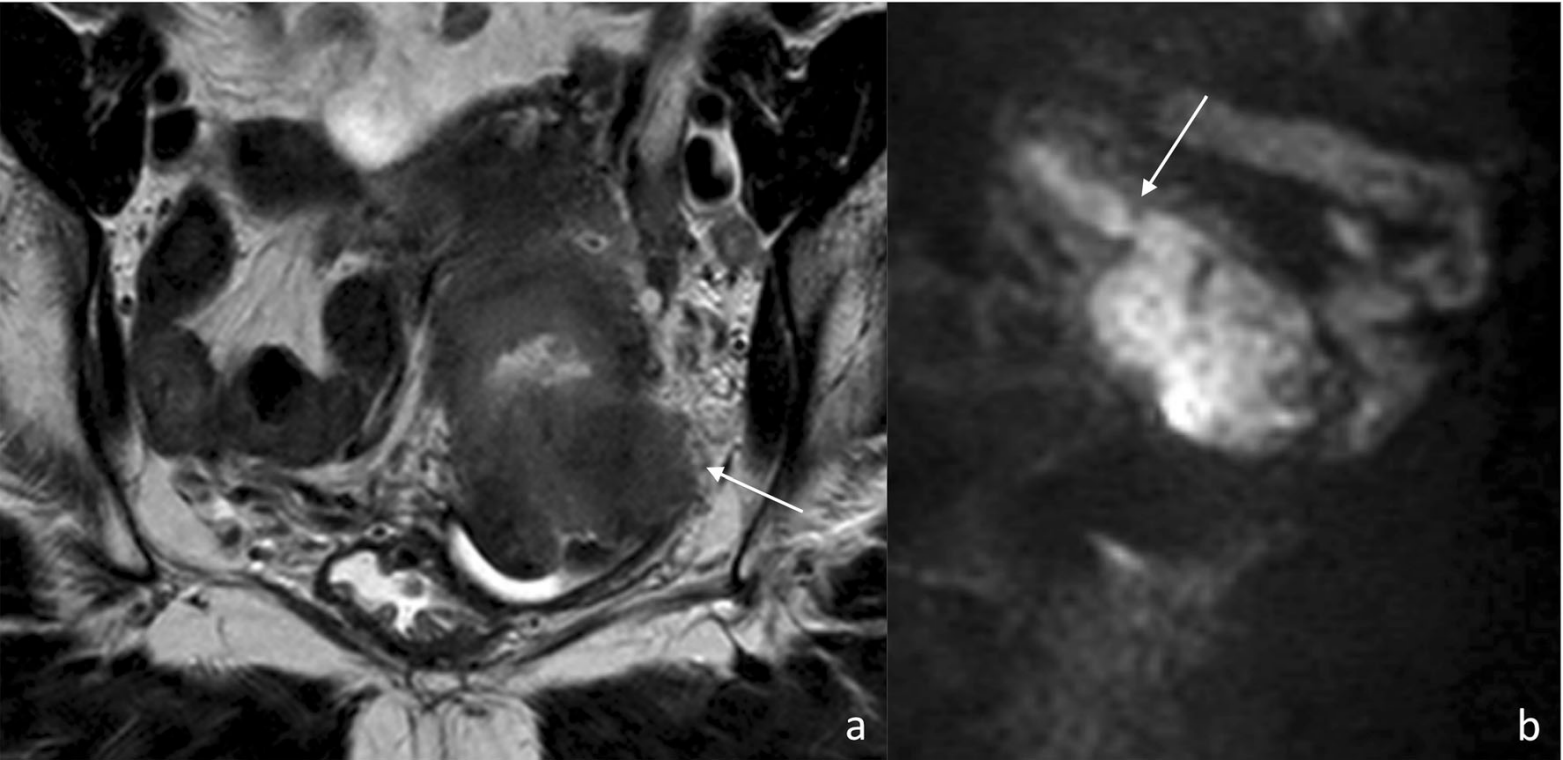
Seventy-two-year-old female patient with stage IIb cervical cancer, as seen on oblique axial high resolution T2-weighted **(a)** and sagittal diffusion-weighted **(b)** images. A bulky cervical mass invades the left subperitoneal mesometrium to its lateral limit, clearly not extending beyond it (*arrow*); therefore, respecting lower Mullerian compartment boundaries **(a)**. In the sagittal plane **(b)**, the mass is seen to invade the uterine corpus (*arrow*), contained in the closely related middle Mullerian compartment

The embryologic development of the Mullerian compartment structures is described in Fig. 4, as well as their subcompartmental distribution. The contents of each subcompartment are given in Table 1 and shown on MR T2-weighted images in Figs. 5, 6, and 7. Examples of local dissemination of tumours contained in the Mullerian compartment are given in Figs. 8 and 9.

**Table 1:** Structures contained in each Mullerian subcompartment [1, 8]

Compartment	Sub- compartments	Structures
Mullerian	Upper	• Fallopian tube except fimbriae
(Anlagen: paramesonephric-mesonephric Mullerian tubercle complex)		• Mesosalpingia
		• Utero-ovarian vascular anastomoses
	Middle	• Uterine corpus
		• Peritoneal mesometria • ligamenta lata
		• Uterine artery and branches and periuterine venous plexus
	Lower	• Uterine cervix
		• Upper 2/3–3/4 of the vagina
		• Subperitoneal mesometria
		• uterine and vaginal vascular pedicles
		• uterovaginal autonomic nerve branches
		• proximal part of utero-sacral ligaments
		• rectovaginal ligaments
		• rectovaginal septum

### Deep urogenital sinus-vaginal complex

**Fig. 10:**
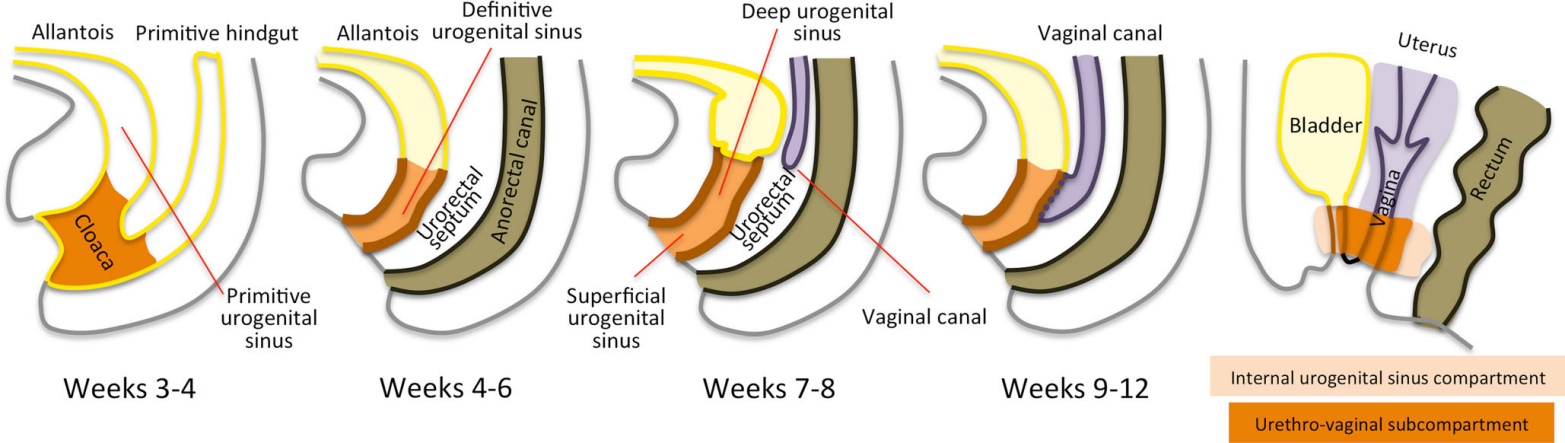
Embryologic development of the distal vagina. **Weeks 3–4:** The distal part of the gastrointestinal tract is in continuity with the allantois. It folds onto itself forming a bulge in the ventral aspect of the embryo—the cloaca; **Weeks 4–6:** A coronal wedge of mesenchyme—the urorectal septum—forms in the angle between the allantois and the hindgut. As it grows caudally, it divides the cloaca into an anterior part—the primitive urogenital sinus—and a posterior part—the anorectal canal; **Weeks 7–8:** The (utero)vaginal canal, which derives from the fused ends of the Mullerian ducts, descends through the urorectal septum; **Weeks 9–12:** Its blind end sinuvaginal eminence comes in contact with the posterior wall of the deep urogenital sinus, inducing the formation of the distal vagina; **Adult:** The structures derived from the deep urogenital sinus form the internal urogenital sinus compartment; they include the urethra, urethrovaginal septum, distal vagina, and distal rectovaginal septum; the posterior wall of the urethra, urethrovaginal septum, and distal vagina form the urethrovaginal subcompartment [1, 12, 14, 32]

**Fig. 11:**
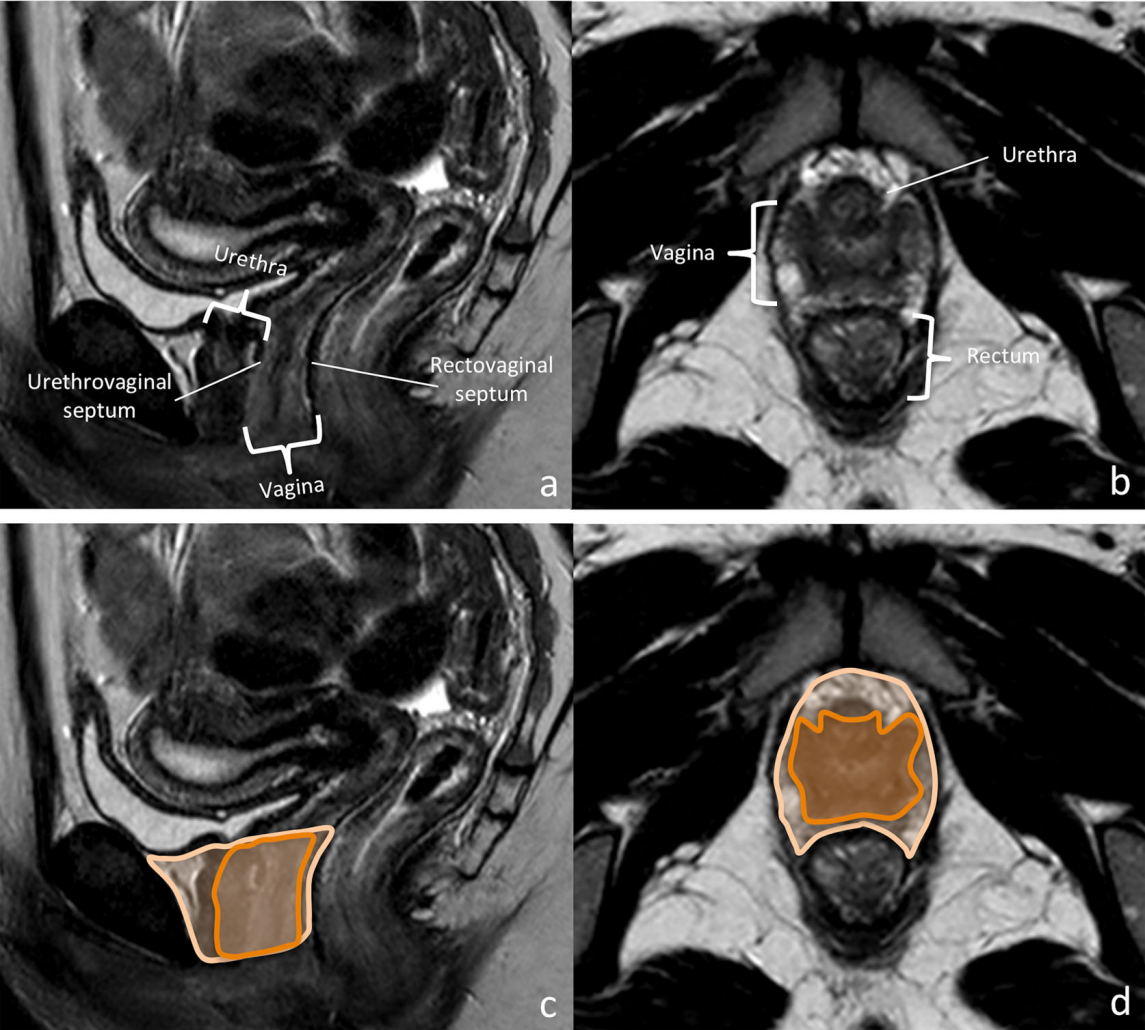
Structures contained in the internal urogenital sinus compartment and compartment boundaries. The internal urogenital compartment is limited anteriorly by the anterior wall of the urethra and posteriorly by the distal rectovaginal septum. It contains the urethrovaginal subcompartment composed of the posterior wall of the urethra, the uretrhrovaginal septum, and distal 1/3 to 1/4 of the vagina. High resolution sagittal (*left*) and axial (*right*) images depict compartment structures (**a** and **b**) and compartment (*light orange*) and subcompartment (*dark orange*) boundaries (**c** and **d**)

The distal vagina develops as a tubulization process in the dorsal wall of the deep urogenital sinus, induced by the descent of the (utero)vaginal canal, which derives from the paramesonephric-mesonephric tubercle complex (Fig. 10). Its formation parallels that of the urethra. These structures and supporting tissue form the internal urogenital sinus compartment. The posterior wall of the urethra, the vaginal wall and rectovaginal septum form a subcompartment within it (Figs. 10 and 11, Table 2) [1, 12, 14].

**Table 2:** Structures contained in the internal urogenital sinus compartment [1, 12, 14]

Compartment	Sub-compartment	Structures
Internal urogenital sinus		
(Anlagen: Deep urogenital sinus-vaginal complex)	Urethrovaginal subcompartment	• Posterior wall of the urethra
		• Urethrovaginal septum
		• Distal vagina
		• Anterior wall of the urethra
		• Distal rectovaginal septum

### Superficial urogenital sinus-genital folds complex

**Fig. 12:**
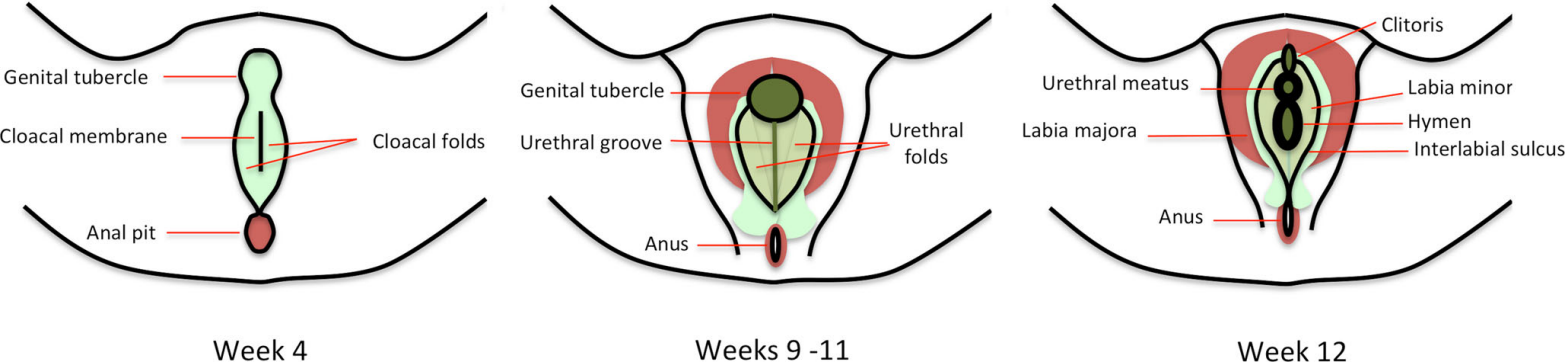
External genitalia formation. **a Week 10**: female differentiation is already apparent. The superficial urogenital sinus-genital folds complex primordium originates the genital tubercle and cloacal folds; **b Week 12**: the urethral folds arise from the cloacal folds and the urethral groove from the cloacal membrane; **c Week 14**: compartments are already formed. Dark green: Inner (vestibular) compartment, composed of the clitoris, urethral meatus, vestibular bulbs, greater vestibular glands, and hymen. Intermediate green: Middle (Glans/labial) compartment, containing the labia minora. Light green: Outer (interlabial) compartment, comprising the interlabial sulcus [1, 13, 14]

The superficial urogenital sinus-genital folds complex is the primordia of the external urogenital sinus compartment, which originates the vulvar structures except the labia majora (Fig. 12). It may be subdivided into three subcompartments, from central to peripheral (Table 3). Compartment boundaries are best depicted under direct observation [1, 13, 14].

**Table 3:** Structures contained in each external urogenital sinus sub-compartment [1, 13, 14]

Compartment	Sub-compartments	Structures
External urogenital sinus	Inner (vestibular)	• Corpus and crura of the clitoris
(Anlagen: Superficial urogenital sinus-genital folds complex)		• Urethral meatus
		• Hymen
		• Vestibular bulbs
		• Greater vestibular glands
	Middle (glans/labial)	• Labia minora
	Outer (interlabial)	• Interlabial sulcus

## Conclusions and future perspectives

Ontogenetic anatomy emerges as an alternative to classic anatomy, focusing on embryologically defined compartments and subcompartments rather than organs and structures. The ontogenetic theory of local tumour spread states cancer is initially confined to the ontogenetic compartment of origin and only after phenotypic changes is able to spread to adjacent compartments, favouring closer embriologic kinship. It has, therefore, had a considerable impact on oncologic surgery. The theory is supported by pattern analysis of various gynaecologic malignancies as well as by improvement in outcomes when ontogenetic anatomy-based surgical procedures are compared to classic techniques. The most dramatic example is colorectal cancer, with the implementation of TME and CME, but data gathered in the past 15 years with respect to gynaecologic malignancies, cervix cancer in particular, has also shown remarkable improvements in local control and disease-free survival. The role of MR imaging for local gynaecologic malignancy staging is, therefore, expected to become as pivotal as in colorectal cancer, but requiring image interpretation from a different perspective. The authors hope that this review has gone some way to introduce these fundamental new concepts to the world of radiology.
